# Adverse childhood experiences and frequent insufficient sleep in 5 U.S. States, 2009: a retrospective cohort study

**DOI:** 10.1186/1471-2458-13-3

**Published:** 2013-01-03

**Authors:** Daniel P Chapman, Yong Liu, Letitia R Presley-Cantrell, Valerie J Edwards, Anne G Wheaton, Geraldine S Perry, Janet B Croft

**Affiliations:** 1 Division of Population Health, National Center for Chronic Disease Prevention and Health Promotion, Centers for Disease Control and Prevention, 4770 Buford Highway NE, Mailstop K-67, Atlanta, GA, 30041, USA

**Keywords:** Insufficient sleep, Childhood abuse, Childhood neglect

## Abstract

**Background:**

Although adverse childhood experiences (ACEs) have previously been demonstrated to be adversely associated with a variety of health outcomes in adulthood, their specific association with sleep among adults has not been examined. To better address this issue, this study examines the relationship between eight self-reported ACEs and frequent insufficient sleep among community-dwelling adults residing in 5 U.S. states in 2009.

**Methods:**

To assess whether ACEs were associated with frequent insufficient sleep (respondent did not get sufficient rest or sleep ≥14 days in past 30 days) in adulthood, we analyzed ACE data collected in the 2009 Behavioral Risk Factor Surveillance System, a random-digit-dialed telephone survey in Arkansas, Louisiana, New Mexico, Tennessee, and Washington. ACEs included physical abuse, sexual abuse, verbal abuse, household mental illness, incarcerated household members, household substance abuse, parental separation/divorce, and witnessing domestic violence before age 18. Smoking status and frequent mental distress (FMD) (≥14 days in past 30 days when self-perceived mental health was not good) were assessed as potential mediators in multivariate logistic regression analyses of frequent insufficient sleep by ACEs adjusted for race/ethnicity, gender, education, and body mass index.

**Results:**

Overall, 28.8% of 25,810 respondents reported frequent insufficient sleep, 18.8% were current smokers, 10.8% reported frequent mental distress, 59.5% percent reported ≥1 ACE, and 8.7% reported ≥ 5 ACEs. Each ACE was associated with frequent insufficient sleep in multivariate analyses. Odds of frequent insufficient sleep were 2.5 (95% CI, 2.1-3.1) times higher in persons with ≥5 ACEs compared to those with no ACEs. Most relationships were modestly attenuated by smoking and FMD, but remained significant.

**Conclusions:**

Childhood exposures to eight indicators of child maltreatment and household dysfunction were significantly associated with frequent insufficient sleep during adulthood in this population. ACEs could be potential indicators promoting further investigation of sleep insufficiency, along with consideration of FMD and smoking.

## Background

Adverse childhood experiences (ACEs) have been associated with a variety of negative behavioral and health outcomes in adulthood [1]. Specifically, exposure to ACEs – which are defined as incidents of household abuse or dysfunction during the first 18 years of life – has been linked to the use of illicit drugs [2], depression [3], psychotropic medication use [4], premature mortality [5], and the prevalence of ischemic heart disease (IHD) [6]. The latter finding was judged noteworthy enough for the authors to conclude that psychological factors, such as anger and depressed affect, appear to be more important than traditional risk factors, including smoking and physical inactivity, in mediating the relation of ACEs to the risk of IHD [6]. Thus, ACEs are powerful events with long-lasting consequences

Furthermore, ACEs have been demonstrated to be highly interrelated to each other. Using data obtained from 8,629 members of a managed healthcare plan who completed a survey about exposure to ACEs, Dong et al. [7] found that the presence of one ACE significantly increased the likelihood of reporting exposure to another ACE. These investigators concluded that the number of respondents observed with an elevated total number of ACEs (the ACE score) was much greater than would be expected given the assumptions of independence, thereby demonstrating that ACEs were statistically interrelated [7].

The effects of ACEs continue long after their initial occurrence. Anda et al. [1] examined the association between the ACE score and the risk of 18 subsequent selected outcomes in affective, somatic, substance abuse, memory, sexual, and aggression-related domains. Notably, a strong, graded relationship emerged between the ACE score and the 18 selected outcomes, leading these investigators to conclude that these relationships parallel the cumulative effect of stress exposure to the developing brain [1].

Likewise, sleep is another event posing serious consequences to health and well-being. It has been estimated that 28% of adults suffer from frequent sleep insufficiency – defined as 14 or more days out of the past 30 that the respondent reported that they did not get enough rest or sleep [8]. In addition to increasing the risk of automobile crashes due to drowsy driving [9], insufficient sleep has been associated with increased body mass index (BMI) [10], obesity [11], diabetes [12], mental distress, depressive symptoms, and anxiety [13].

Notably, ACEs have been linked to sleep disturbances among adults. In a retrospective cohort study of health maintenance organization members in California which examined respondents’ reports of ever having trouble falling or staying asleep and feeling tired even after a good night’s sleep, Chapman et al. [14] found that ACEs were associated with both types of reported sleep disturbances in adulthood, and that the ACE score assumed a graded relationship to them. However, these results were obtained from a large managed care patient population which, while reasonably representative of the demographics of the sample investigated, may nonetheless differ from the population at large. Thus, this investigation examines the relationship between ACEs and frequent insufficient sleep among adults in a more diverse population-based sample.

Both smoking [13,15] and frequent mental distress [13] are associated with insufficient sleep. Therefore, we present models controlling for these variables to better elucidate the relationship between ACEs and sleep and to determine if ACEs could serve, potentially, as indicators promoting the further investigation of sleep sufficiency.

## Methods

### Data selection

Data were obtained from the 2009 Behavioral Risk Factor Surveillance Survey (BRFSS), a state-based random-digit dialed telephone survey of non-institutionalized U.S. adults aged 18 years and older. Trained interviewers administered standardized questionnaires to a sample of households with landline telephones. Five states (Arkansas, Louisiana, New Mexico, Tennessee, and Washington) administered a supplemental ACE survey question module as part of the BRFSS survey. In the five states investigated, cooperation rates – the proportion of all respondents interviewed -- ranged from 70.1% (Washington) to 78.8% (Arkansas) [16]. The BRFSS survey was approved by the Institutional Review Boards of the Centers for Disease Control and Prevention and all participating states’ Departments of Health. During the telephone interview, all respondents provided informed consent to participate in the BRFSS survey. Of 29,212 respondents in these 5 states, 25,810 (88.4%) of adults aged 18 years or older completed all questions pertaining to ACEs and perceived number of days of insufficient sleep. We excluded respondents with missing values (6.0%) or refusals (4.2%) on one or more of the ACE questions, as well as respondents with missing values on the question pertaining to sufficient sleep (1.4%).

### Frequent insufficient sleep

The outcome variable of interest, frequent insufficient sleep, was defined as a response of ≥14 days to the following question, “During the past 30 days, for about how many days have you felt you did not get enough rest or sleep?”

### Adverse childhood experiences

The eleven questions included about ACEs were adopted from a longer set of ACE-related questions featured in a study, conducted jointly by the Kaiser Permanente Health Appraisal Clinic in San Diego, CA and the Centers for Disease Control and Prevention, to assess associations between childhood maltreatment and well-being later in life [17]. Questions comprising the 2009 ACE module on the BRFSS, measured three categories of childhood abuse (verbal, physical, and sexual abuse) and five categories of household dysfunction (household mental illness, incarcerated household members, household substance abuse, parental separation/divorce, and witnessing domestic violence) to which the respondent was exposed during childhood. Alternative responses to the questions were provided by the interviewer to the respondent. Notably, less than 0.5% responded “don’t know or not sure” to any one of the ACE questions, which was defined as a negative response for the ACE category referenced [18].

Verbal abuse was defined as a response of “more than once” to the question “How often did a parent or adult in your home ever swear at you, insult you, or put you down?”. Physical abuse was defined as a response of either “once” or “more than once” to the question “How often did your parents or adult in your home ever hit, beat, kick, or physically hurt you in any way? Do not include spanking.” A response of either “once” or “more than once” to any of the following three questions defined sexual abuse: “How often did anyone at least 5 years older than you or an adult, ever touch you sexually?”, “How often did anyone at least 5 years older than you or an adult try to make you touch them sexually?” or “How often did anyone at least 5 years older than you or an adult force you to have sex?”

Affirmative responses to questions about living with a household member who “was depressed, mentally ill, or suicidal” or who “served time or was sentenced to serve time in prison, jail, or other correctional facility” defined two respective household dysfunction variables. Living with a household substance abuser was defined as a “yes” response to at least one of two questions about living with anyone who “was a problem drinker or alcoholic” or “used illegal street drugs or abused prescription medications.” Having “parents who were separated or divorced” was defined by a “yes” response to a question asking about that as opposed to answering “no,” or “parents not married.” A response of “once” or “more than once” to the question “How often did your parents or adults in your home ever slap, hit, kick, punch, or beat each other up” defined witnessing domestic violence [18].

The ACE score was created by summing the total number of ACE categories which elicited positive responses (range: 0-8). ACE scores of 5 or more were combined into one category (≥5) due to small sample sizes. Six categories of ACE score (0, 1, 2, 3, 4, and ≥5) were featured in analyses, with zero experiences serving as the referent. Specifically, ≥5 ACEs were combined into a single category to obtain adequate statistical power and because of the consistently graded relationship observed between frequent insufficient sleep and high (≥5) ACE score (data not shown).

### Potential mediators

Smoking status was defined as current smokers (smoked ≥100 cigarettes in the past and were smoking every day or some days now), former smokers (smoked ≥100 cigarettes in the past, but were not smoking now), or non-smokers (those who never smoked). Respondents were asked to report their perceived mental health in a question gauging frequent mental distress (FMD). Respondents were considered as having FMD if they answered ≥14 days to the question, “Now thinking about your mental health, which includes stress, depression, and problems with emotions, for how many days during the past 30 days was your mental health not good?”

### Covariates

Gender, age in years (18-24, 25-34, 35-44, 45-64 and ≥65), race/ethnicity (non-Hispanic white, non-Hispanic black, Hispanic, or other non-Hispanic), education (less than high school graduate, high school diploma or GED, some college, or college graduate), and body mass index in kg/m^2^ (BMI <18.5 was defined as underweight, BMI=18.5-24.9 as normal weight, BMI=25.0-29.9 as overweight, and BMI ≥30 as obese) were examined as covariates in the association between ACEs and frequent insufficient sleep.

### Statistical analyses

Adjusted odds ratios (AORs) and 95% confidence intervals (CIs) for frequent insufficient sleep by individual ACE and by ACE score were obtained from multivariate logistic regression models adjusting for gender, age, race/ethnicity, education, and BMI, as well as the potential mediators of smoking status or FMD. We assessed whether the relationship between ACEs and days of insufficient sleep was attenuated by smoking status or FMD by measuring the percentage of change in odds ratios evidenced between a model with and a model without the specific mediator = [(OR _no mediator_ - OR _with mediator_ )/(OR _no mediator_ -1)] * 100 [19,20]. To be statistically conservative, 10% or more of the percentage change indicated that there was a significant mediation effect [19] only if the following three criteria were met: 1) there was a statistically significant relationship between ACEs and frequent insufficient sleep, 2) there was a significant relationship between ACEs and the mediating variable, and 3) there was a significant relationship between the mediating variable and frequent insufficient sleep [21].

All analyses were conducted using SAS-callable SUDAAN to account for the complex study design, with a statistical significance level of p<0.05.

## Results

### Study population characteristics

The study population was comprised of 16,474 women (52%) and 9,336 men (48%) from five U.S. states (Table 1). The majority of the study population was non-Hispanic white (77.1%) and had at least some college education (62.9%). Sixty-six percent (66.3%) of respondents were overweight or obese, and 18.8% were current smokers. Frequent mental distress was reported by 10.8% of respondents and 28.8% reported frequent sleep insufficiency.

**Table 1:** Selected participant characteristics, including frequent insufficient sleep, individual ACEs, and ACE score among adults aged 18 years and older in 5 states, 2009 BRFSS

**Characteristic**	**Unweighted sample size**	**Weighted % (95% CI)**
**Total**	25,810	100.0
**Gender**		
Men	9,336	48.4 (47.3-49.6)
Women	16,474	51.6 (50.4-52.7)
**Age, years**		
18-24	750	8.1 ( 7.3- 8.9)
25-34	2,073	17.2 (16.2-18.2)
35-44	3,354	23.3 (22.1-24.4)
45-54	5,300	19.5 (18.7-20.3)
55-64	6,062	15.1 (14.6-15.7)
≥65	8,140	16.8 (16.2-17.4)
**Race/ethnicity**		
White, Non-Hispanic	19,485	77.1 (76.2-78.0)
Black, Non-Hispanic	2,599	10.7 (10.0-11.5)
Hispanic	2,179	6.7 ( 6.3- 7.2)
Non-Hispanic/Others*	1,356	5.4 ( 4.9- 5.9)
**Education attainment**		
<High school	2,540	9.0 ( 8.3- 9.7)
High school graduates or GED	7,236	28.1 (27.0-29.1)
Some college	7,465	30.1 (29.0-31.1)
College graduates	8,541	32.8 (31.9-33.8)
**Body Mass Index, kg/m**^**2**^		
Underweight (<18.5)	407	1.6 ( 1.3- 1.9)
Normal Weight (18.5-24.9)	8,110	32.1 (31.0-33.1)
Overweight (25.0-29.9)	8,800	35.4 (34.3-36.6)
Obese (≥30)	7,541	30.9 (29.8-32.0)
**Smoking status**		
Current smokers	4,336	18.8 (17.8-19.7)
Former smokers	7,748	26.2 (25.2-27.2)
Never smoked	13,639	55.0 (53.9-56.1)
**Frequent mental distress (≥14 days/the past 30 days)**	2,752	10.8 (10.1-11.6)
**ACEs before age 18**		
Verbal abuse	6,297	26.1 (25.1-27.0)
Physical abuse	3,616	14.8 (14.0-15.6)
Sexual abuse	3,310	12.1 (11.4-12.7)
Substance abusing household member	7,021	29.1 (28.1-30.2)
Household mental illness	4,423	19.5 (18.6-20.4)
Witnessing domestic violence	3,906	16.3 (15.5-17.2)
Incarcerated household member	1,261	7.2 ( 6.4- 7.9)
Parental separation/divorce	5,583	26.6 (25.6-27.7)
**ACE score**		
0	11,208	40.5 (39.4-41.6)
1	5,894	22.4 (21.5-23.4)
2	3,221	13.1 (12.3-13.9)
3	2,179	8.8 ( 8.2- 9.4)
4	1,435	6.5 ( 5.9- 7.2)
≥5	1,873	8.7 ( 8.0- 9.3)
**Frequent insufficient sleep (≥14 days/the past 30 days)**	6,522	28.8 (27.7-29.8)

*Non-Hispanic/others includes persons responding as non-Hipanic and Asian, Native Hawaiian or Pacific Islander, American Indian or Alaska Native, Multiracial, or other race only.

The majority of respondents reported ≥1 ACE (59.5%) and 8.7% reported ≥5 ACEs. Specifically, the most frequently reported ACEs were having lived with a substance abusing household member (29.1%), parental separation or divorce (26.6%), and exposure to verbal abuse (26.1%) during childhood (Table 1).

### Frequent insufficient sleep

As evident in Table 2, a greater percentage of frequent insufficient sleep was reported by women than men (30.6% vs. 26.8%, respectively, p <0.05), among respondents aged 25-34 years relative to those aged ≥65 years (35.4% vs. 15.4%, respectively, p <0.05). Frequent insufficient sleep was likewise more prevalent among those with less than a high school education than among college graduates (35.4% vs. 25.1%, respectively, p <0.05), and among those who were obese than among those of normal weight (33.2% vs. 26.6%, respectively, p <0.05).

**Table 2:** Prevalence of insufficient sleep (≥14 days/30 days) by selected participant characteristics, individual ACEs and ACE score among adults aged ≥18 years in 5 states, 2009 BRFSS

**Characteristic**	**% (95% CI)**
**Total**	28.8 (27.7-29.8)
**Sex**	
Men	26.8 (25.1-28.6)
Women	30.6 (29.4-31.8)
**Age, years**	
18-24	32.3 (27.6-36.9)
25-34	35.4 (32.2-38.6)
35-44	33.6 (30.8-36.3)
45-54	30.3 (28.3-32.2)
55-64	25.3 (23.5-27.0)
≥65	15.4 (14.2-16.5)
**Race/ethnicity**	
White, Non-Hispanic	28.4 (27.2-29.6)
Black, Non-Hispanic	31.5 (27.9-35.0)
Hispanic	26.4 (23.4-29.5)
Others	32.6 (28.2-37.1)
**Educational attainment**	
<high school	35.4 (31.4-39.4)
High school graduates or GED	27.7 (25.7-29.7)
Associate degree	31.9 (29.8-34.0)
College graduates	25.1 (23.5-26.7)
**Body Mass Index, kg/m**^**2**^	
Underweight (<18.5)	33.5 (25.1-41.8)
Normal Weight (18.5-24.9)	26.6 (24.7-28.5)
Overweight (25.0-25.9)	27.0 (25.2-28.8)
Obese (≥30)	33.2 (31.2-35.2)
**Heavy drinking**	
No	28.6 (27.5-29.7)
Yes	31.7 (27.2-36.3)
**Smoking status**	
Current smokers	40.3 (37.3-43.2)
Former smokers	27.3 (25.4-29.3)
Never smoked	25.5 (24.2-26.9)
**Frequent mental distress (≥14 days/the past 30 days)**	
No	24.5 (23.5-25.6)
Yes	63.2 (59.6-66.8)

### Relationships between potential mediators and frequent insufficient sleep

Frequent insufficient sleep was more prevalent among current smokers than among those who had never smoked (40.3% vs. 25.5%, respectively, p <0.05). Frequent insufficient sleep was also higher among those reporting FMD than among those who did not (63.2% vs. 24.5%, respectively, p <0.05) (Table 2).

### Relationships between adverse childhood experiences and potential mediators

Current smoking and FMD were significantly higher (p <0.05) among persons with each individual ACE than among those without that ACE (Table 3). Smoking increased with the ACE score such that the prevalence of current smoking was 37.8% among those with ≥5 ACEs compared to 13.0% among those with no ACEs (p <0.05). FMD also increased with increasing ACE score such that the prevalence of FMD was 27.3% among those with ≥5 ACEs compared to 6.0% among those with no ACEs (p <0.05).

**Table 3:** Prevalence of current smoking and frequent mental distress by individual ACEs and ACE score, 2009 BRFSS

**Adverse childhood experience**	**Current smoking % (95% CI)**	**Frequent mental distress % (95% CI)**
**Verbal abuse**		
No	16.1 (15.0-17.2)	8.2 ( 7.4- 9.0)
Yes	26.4 (24.4-28.4)	18.2 (16.5-20.0)
**Physical abuse**		
No	17.1 (16.1-18.1)	9.1 ( 8.3- 9.9)
Yes	28.5 (25.8-31.2)	21.1 (18.7-23.4)
**Sexual abuse**		
No	17.6 (16.6-18.6)	9.4 ( 8.6-10.2)
Yes	27.4 (24.5-30.3)	21.2 (18.8-23.6)
**Substance abusing household member**		
No	15.5 (14.5-16.6)	8.3 ( 7.5- 9.1)
Yes	26.7 (24.6-28.8)	17.0 (15.3-18.7)
**Mentally ill household member**		
No	17.4 (16.3-18.4)	8.8 ( 8.0- 9.6)
Yes	24.6 (22.4-26.9)	19.3 (17.3-21.3)
**Witnessing domestic violence**		
No	17.1 (16.1-18.0)	9.2 ( 8.4- 9.9)
Yes	27.7 (24.7-30.6)	19.4 (16.9-21.9)
**Incarcerated household member**		
No	17.3 (16.4-18.3)	9.9 ( 9.2-10.6)
Yes	37.5 (32.0-43.0)	22.9 (18.4-27.4)
**Parental separation/ divorce**		
No	15.8 (14.8-16.7)	9.6 ( 8.8-10.4)
Yes	27.1 (24.7-29.5)	14.2 (12.3-16.1)
**ACE score**		
0	13.0 (11.7-14.2)	6.0 ( 5.1- 6.9)
1	17.9 (15.9-19.9)	8.9 ( 7.2-10.5)
2	19.5 (16.6-22.4)	11.1 ( 9.3-12.9)
3	21.4 (18.2-24.6)	16.8 (13.5-20.0)
4	27.8 (23.0-32.5)	17.2 (13.8-20.6)
≥5	37.8 (33.7-41.9)	27.3 (23.6-30.9)

### Relationships between adverse childhood experiences and frequent insufficient sleep

Respondents reporting each individual ACE had a significantly greater prevalence of frequent sleep insufficiency relative to respondents who indicated they had not been exposed (p <0.05) (Table 4). Similarly, the ACE score assumed a significant graded relationship with the prevalence of frequent sleep insufficiency. Among those indicating no ACEs, 21.3% reported frequent insufficient sleep relative to 47.0% of those with ≥5 ACEs (p <0.05). This association remained significant after adjusting for sex, age, race/ethnicity, education, and categorical BMI (adjusted odds ratio [AOR]=2.53; 95% confidence interval [CI]=2.09-3.06) (Table 4, Model 1).

**Table 4:** Crude prevalence and adjusted odds ratio (OR) and 95% confidence interval of frequent insufficient sleep (≥14 days/30 days) by individual ACEs and ACE score, 2009 BRFSS

**ACE**	**% (95% CI)**	**Model 1 AOR (95% CI)**	**Model 2 AOR (95% CI)**	**Model 3 AOR (95% CI)**
**Verbal abuse**				
No	25.6 (24.4-26.8)	1.00 (referent)	1.00 (referent)	1.00 (referent)
Yes	37.9 (35.8-40.0)	1.59 (1.42-1.79)	1.50 (1.33-1.68)*	1.32 (1.16-1.50)†
**Physical abuse**				
No	26.7 (25.6-27.8)	1.00 (referent)	1.00 (referent)	1.00 (referent)
Yes	40.9 (38.0-43.8)	1.71 (1.50-1.97)	1.62 (1.41-1.86)*	1.43 (1.23-1.65)**
**Sexual abuse**				
No	27.1 (26.0-28.3)	1.00 (referent)	1.00 (referent)	1.00 (referent)
Yes	40.9 (37.9-43.8)	1.66 (1.44-1.93)	1.56 (1.35-1.81)*	1.40 (1.19-1.64)**
**Substance abusing household member**				
No	25.7 (24.5-26.8)	1.00 (referent)	1.00 (referent)	1.00 (referent)
Yes	36.4 (34.3-38.6)	1.47 (1.30-1.65)	1.38 (1.23-1.55)*	1.25 (1.10-1.42)†
**Mentally ill Household member**				
No	26.0 (24.8-27.1)	1.00 (referent)	1.00 (referent)	1.00 (referent)
Yes	40.4 (37.9-43.0)	1.68 (1.47-1.92)	1.60 (1.40-1.83)*	1.42 (1.23-1.64)**
**Witnessing domestic violence**				
No	26.8 (25.7-27.9)	1.00 (referent)	1.00 (referent)	1.00 (referent)
Yes	39.1 (36.2-42.0)	1.58 (1.37-1.82)	1.51 (1.31-1.73)*	1.35 (1.16-1.57)**
**Incarcerated household member**				
No	27.8 (26.7-28.8)	1.00 (referent)	1.00 (referent)	1.00 (referent)
Yes	42.1 (36.7-47.5)	1.54 (1.21-1.95)	1.40 (1.10-1.78)**	1.23 (0.95-1.60)†
**Parental separation/ divorce**				
No	26.5 (25.4-27.7)	1.00 (referent)	1.00 (referent)	1.00 (referent)
Yes	35.0 (32.6-37.3)	1.23 (1.08-1.40)	1.16 (1.02-1.32)**	1.14 (0.99-1.31)**
**ACE score**				
0	21.3 (19.8-22.8)	1.00 (referent)	1.00 (referent)	1.00 (referent)
1	27.0 (24.9-29.1)	1.28 (1.11-1.49)	1.25 (1.08-1.45)*	1.21 (1.04-1.41)**
2	34.7 (31.6-37.8)	1.74 (1.46-2.06)	1.68 (1.41-1.99)	1.61 (1.35-1.92)*
3	34.4 (31.0-37.8)	1.68 (1.40-2.02)	1.60 (1.33-1.93)*	1.41 (1.15-1.72)**
4	38.1 (33.0-43.2)	1.91 (1.48-2.46)	1.77 (1.39-2.26)*	1.56 (1.20-2.03)**
≥5	47.0 (42.9-51.0)	2.53 (2.09-3.06)	2.26 (1.86-2.75)*	1.80 (1.46-2.21)†

Model 1: multivariate logistic regression model with sex, age, race/ethnicity, education, and categorical BMI.

Model 2: Model 1 plus smoking status (current smoking vs. former smoker/never smoked).

Model 3: Model 1 plus FMD.

*-10%-<20% percentage change of OR in term of formula=((OR_1_-OR_2(3)_)/(OR_1_-1.0))*100.

** 20%-<40% percentage change of OR.

†40%-<60% percentage change of OR.

### Mediating effects

The effects of variables potentially mediating the relationship between ACEs and frequent sleep insufficiency are presented in Table 4 (Models 2 and 3). Model 2 adjusts for all covariates included in Model 1 with the addition of smoking status. All of the individual ACEs remained significantly associated with frequent sleep insufficiency, although the relationship between ACEs and sleep insufficiency was slightly attenuated by the addition of smoking status to the model. The ACE score retained a significant graded relationship with frequent insufficient sleep ([AOR (95% CI)=1.25 (1.08-1.45) for 1 ACE and =2.26 (1.86-2.75) for ≥5 ACEs] relative to persons with no ACEs). The attenuation observed suggests that smoking status may be a partial mediator of the relationship between ACEs and frequent sleep insufficiency (Table 4, Model 2).

Similarly, Model 3 includes all covariates featured in Model 1, while additionally adjusting for FMD. Further attenuation of many of the ACEs is observed, which is consistent with partial mediation of the relationship between ACEs and frequent insufficient sleep. However, frequent insufficient sleep was no longer significant for the associations with an incarcerated household member (AOR=1.23[0.95-1.60]), or parental divorce/separation (AOR=1.14[0.99-1.31]) with the addition of FMD to the model. This suggests that FMD exerted a greater effect on frequent sleep insufficiency than these two ACEs. A graded -- albeit further attenuated -- relationship was observed between the ACE score and frequent sleep insufficiency: AOR for 1 ACE=1.21 (1.04-1.41), while AOR for ≥5 ACEs=1.80 (1.46-2.21) compared to persons with no ACEs (Table 4, Model 3).

## Discussion

This investigation indicates that ACEs experienced before age 18 are positively associated with frequent insufficient sleep in adult community dwellers. As previously noted, these results are similar to those from prior research examining the association between ACEs and two forms of disturbed sleep in a managed care patient population [14]. The current findings thus extend this association of ACEs with sleep impairment to specifically include frequent insufficient sleep within the domain of a population-based sample.

Although associations between childhood sexual abuse and sleep disturbance have been previously reported [22-24], this research has largely been restricted to investigations featuring relatively brief follow-up intervals [23], or only gauging childhood sexual abuse [22-24]. However, related investigation has found both childhood sexual abuse and childhood physical abuse to be positively associated with difficulties falling asleep, staying asleep, or early awakening among a cohort of individuals with a median age of 47 years [25]. This finding is consistent with the results of previous research revealing these forms of childhood abuse were associated with sleep disturbance decades after their occurrence [14].

In an investigation assessing the frequency of self-reported childhood physical, emotional, and sexual abuse, Greenfield, Lee, Friedman, and Springer [26] analyzed data from 835 respondents in the National Survey of Midlife Development in the United States. Specifically, these investigators examined the association of these three forms of abuse with global sleep pathology, as measured by the Pittsburgh Sleep Quality Inventory, as well as with a variety of specific sleep characteristics, such as subjective sleep quality, sleep duration, and habitual sleep efficiency in adulthood. Consistent with the results of the present investigation, that study reported that all three types of childhood abuse examined were associated with global sleep pathology, as well as with specific sleep problems [26]. Additionally, related investigation has revealed that childhood adversities such as parental divorce, long-term financial difficulties, and frequent fear of a family member were associated with poor self-reported sleep quality in adulthood [27].

Notably, the results of our investigation are similar to those obtained among a clinical sample. Bader, Shafer, Schenkel, Nissen, and Schwander [28] examined the association of ACEs with actigraphic and polysomnographic sleep parameters. These investigators found that patients who reported moderate to severe ACEs exhibited a significantly greater number of both awakenings and movement arousals relative to patients reporting few or no ACEs.

The present study further addressed the effects of smoking and FMD on the associations between ACEs and frequent insufficient sleep. These relationships were slightly attenuated by smoking status and moderately attenuated by FMD. Our study findings are consistent with the results of research indicating that smoking is significantly associated with insufficient sleep [13,15,29]. Further, smoking is associated with short and long sleep durations, both of which have been linked to risk for excess mortality [30].

The attenuation of the association between ACEs and frequent insufficient sleep by FMD is consistent with psychiatric nomenclature featuring sleep disturbance as a criterion for the diagnosis of depression [31]. Early detection of and intervention regarding ACEs thus appear particularly warranted as insomnia symptoms [32] and sleep disturbance [33] have been reported to persist even after depression has improved or remitted. Notably, childhood abuse has also been associated with posttraumatic stress disorder (PTSD) symptoms [34], with recent evidence suggesting that the nightmares and insomnia characteristic of PTSD may compromise the consolidation of fear extinction memory and, ultimately, recovery [35]. This further suggests the potentially lasting impact of ACEs, although PTSD was not assessed in this investigation.

This investigation is subject to several limitations. First, our data on both sleep and ACEs were obtained by self-report, with the former assessed by a single item not corroborated by actigraphy, polysomnography, or medical records, Specifically, relative to data garnered by objective measures, sleep difficulties are frequently overreported [36]. However, previous research examining potential biases in the elicitation of ACEs concluded that retrospective self-reports of childhood abuse or neglect are apt to be conservative [37], thereby biasing the results towards the null. Second, our results are cross-sectional thereby prohibiting any inference of causality. Moreover, the presence of ACEs might also increase the risk of adverse events after age 18 years, which this data set does not permit assessment of. Additonally, given that the definition of FMD describes behavioral characteristics not inconsistent with those associated with ACEs, the association between these phenomena could be circuitous. This study was also limited to participants with landline telephones. Subsequent research has indicated that, relative to adults with landlines, adults with only cell phones are more likely to be binge drinkers and current smokers, to engage in regular physical activity and to have an unmet medical care need due to cost, and to be less likely to be use preventive healthcare services, and to be obese [38]. Finally, the generalizability of our findings may be limited, as data were collected from five states.

## Conclusion

In conclusion, the results of the present investigation indicate that ACEs are positively associated with frequent insufficient sleep in U.S. adult community-dwellers. This relationship was attenuated – but generally remained significant – by FMD and smoking. These results suggest that ACEs could be potential indicators fostering further investigation of sleep insufficiency, along with consideration of FMD and smoking.

## Abbreviations

ACE: Adverse childhood experiences; BMI: Body mass index; BRFSS: Behavioral risk factor surveillance survey; FMD: Frequent mental distress; GED: General education diploma.

## Competing interests

The authors declare that they have no competing interests.

## Authors’ contributions

DC, JC, YL all have made substantial contributions to the conception, design, and acquisition of data. AW, DC, JC, GP, LPC, VE all has made substantial contributions to the analysis and interpretation of data. DC, JC, YL have been involved in drafting the manuscript; AW, DC, GP, JC, LPC, VE have been involved in critically revising the manuscript for important intellectual content. All co-authors have read and approved this manuscript.

## Disclaimer

The findings and conclusions in this report are those of the authors and do not necessarily represent the official position of the Centers for Disease Control and Prevention or the Association for Prevention Teaching and Research.

## Sources of support

AGW received support from a fellowship through a cooperative agreement (award number 3U50CD300860) between the Association for Prevention Teaching and Research and the Centers for Disease Control and Prevention. This project was supported, in part, by cooperative agreement U58/CCU324336-05 between the National Association of Chronic Disease Directors and the Centers for Disease Control and Prevention.

## Pre-publication history

The pre-publication history for this paper can be accessed here:

http://www.biomedcentral.com/1471-2458/13/3/prepub
